# Exploring brain activity and transforming knowledge in visual and textual programming using neuroeducation approaches

**DOI:** 10.3934/Neuroscience.2019.3.175

**Published:** 2019-09-02

**Authors:** Spyridon Doukakis

**Affiliations:** Department of Informatics, Ionian University, 7 Tsirigoti Square, 49132 Corfu, Greece

**Keywords:** programming, EEG, novice programmers, undergraduate students, learning, knowledge, transformation

## Abstract

Eight (8) computer science students, novice programmers, who were in the first semester of their studies, participated in a field study in order to explore potential differences in their brain activity during programming with a visual programming language versus a textual programming language. The eight students were asked to develop two specific programs in both programming languages (a total of four tasks). The order of these programs was determined, while the order of languages in which they worked differed between the students. Measurement of cerebral activity was performed by the electroencephalography (EEG) imaging method. According to the analysis of the data it appears that the type of programming language did not affect the students' brain activity. Also, six students needed more time to successfully develop the programs they were asked with the first programming language versus the second one, regardless of the type of programming language that was first. In addition, it appears that six students did not show reducing or increasing brain activity as they spent their time on tasks and at the same time did not show a reduction or increase in the time they needed to develop the programs. Finally, the students showed higher average brain activity in the development of the fourth task than the third, and six of them showed higher average brain activity when developing the first versus the second program, regardless of the programming language. The results can contribute to: a) highlighting the need for a diverse educational approach for students when engaging in program development and b) identifying appropriate learning paths to enhance student education in programming.

## 1. Introduction

Computer Science students, in the first semester of their studies, learn programming through relevant courses. According to the existing research, it has been observed that the students have difficulties to cope with some of the demands of programming courses, which has as a result to avoid getting deeper into programming [1],[2]. For this reason, different and multiple approaches to introduce programming have been proposed to enable students to better understand and progress as programmers [3]. The research has contributed to the improvement of programming courses, modification of teaching approaches and have led to educational interventions [4],[5].

At the same time, neuroscience attempts to explain how the brain and mind work together by exploring brain and nervous system functioning. The field contributes to our basic understanding of the neural mechanisms that form the basis for human development and learning. These efforts attempt to link neuroscience with cognitive science, psychology and education and have led to the development of a new interdisciplinary field titled Neuroeducation. In this new field educational experts, neuroscientists and cognitive scientists collaborate to implement the findings of neuroscientific research in educational contexts. Recent advances in brain imaging techniques, have provided additional opportunities for researchers to explore the functional organization of the human brain [6]–[9].

The aim of this research is to explore possible differences in the brain activity of eight computer science students with the electroencephalogram (EEG) method. EEG is generally an noninvasive method to record electrical activity of the brain [10]. In the present paper, a brief overview of the research field of neuroscience, brain development and brain imaging are presented. The paper continues with the research methodology and the results according to the analysis of the qualitative and quantitative data collected. The work ends with a discussion of conclusions and proposals for further research.

## 2. Teaching computer programming

Computer programming courses are included in all computer science departments curricula. Moreover, because computer programming is also the passage from theoretical to applied computer science [11], computer programming course can be found as a distinct subject or as part of other courses. Additionally, in the market, computer programming knowledge is an important qualification. The above-mentioned observations could positively influence the learner's choices and lead them to study and graduate from special programs or higher education departments that will give them the basis to work as professionals in the field of computer programming. However, research shows that several students of computer science departments or adults that attend special programs on computer programming have difficulties, which is leading to high dropout rates from the courses. Students' difficulties may be related with problem understanding, its decomposition, algorithm creation using a flow chart or pseudocode and finally with the implementation of the solution in a programming language [12].

The above difficulties may be associated with the absence of appropriate mental models of programming concepts and/or the absence of problem-solving ability. These difficulties lead to obstacles and misunderstandings of learners both in the learning of programming concepts and also coding. These obstacles appear when learners study the syntax of different programming languages, try to understand code sections, track and debug code fragments [13]–[16].

The above highlights the complexity of programming teaching and the related challenge that emerges in the field of computer science teaching [16]. Researchers such as Pears et al. have recorded a) the curriculum, b) the pedagogical approach, c) the programming language that will be used, and d) the tools that will support the course as the four categories that play a key role in building programming courses [17]. At the same time, a significant number of surveys have focused on teaching approaches, where researchers have made proposals that either arise from their own beliefs and their own experiences or from the market needs [18]–[20].

Other studies highlight the importance of appropriate representations and argue that learner knowledge is enhanced when interact through appropriate representations [21]. In programming, the use of different representations is inherent in the various stages of program development. There are different ways of algorithm representations (eg diagrammatic techniques, pseudocode, algorithmic steps) and different program development representations (eg textual and visual programming) [22]. In this context, the training of future developers focuses also on the different representations of program development. In particular, learners come in contact with both textual and visual programming, using suitable programming languages. These two types of programming are also dominant in the labor market. In this way, future developers are trained in both programming approaches and are able to choose the appropriate type of programming depending on the application they are required to develop (eg mobile applications).

Existing research attempts to study issues related to the design of relevant learning curricula for the learning of different types of programming and the impact these programs have on education and learning of prospective developers [5],[21],[23]–[25]. One of the important research questions is the study of the cognitive process that takes place during programming. Existing research explores general cognitive theories of problem-solving and evaluates cognitive processes. The research is focused on structure and semantics of information, the acquisition of knowledge, the construction of knowledge, and the design of solutions [26]. To achieve this, researchers choose to make experiments in order to test a hypothesis with groups of trainees. The groups work with the same activities and the researchers observe the trainees and measure both the duration and the accuracy of the implementation of the activities. In recent years, research has exploited triangulation techniques, long-term studies and long-term learner tracking [23].

From the above, it seems that the way of teaching programming is an important issue for both the labor market and the quality of computer programming training programs. The present study tries to contribute to this area, using brain imaging techniques in order to record students' brain activity during programming.

## 3. Neuroeducation, brain activity and computer programming

The collaboration of neuroscientists, cognitive scientists, psychologists and educators has led to the creation of a new interdisciplinary field which is called neuroeducation. It aims to contribute initially to the understanding and then to the improving learning. Neuroeducation, “better reflects a field with education at its core, uniquely characterized by its own methods and techniques, and which constructs knowledge based on experiential, social and biological evidence” [9],[27]. Although the results of the research in neuroeducation, cannot translate directly into teaching practices and specific learning pathways, they can contribute to learning [28]. With the help of the results, it can be described what the trainees can do, but it is not possible to determine what the trainees could do in the learning process [27],[29].

For these reasons, recording brain activity offers the possibility to identify the neurodevelopmental differences that affect educational results and identify individual differences in the trainees' brain that contribute to reflecting the level of learning according to the curriculum [30]. With the collection of relevant data, specialists have the opportunity to modify and create differentiated curricula, and at the same time educators have the opportunity to review or modify their teaching practices and lead them to work on the creation of teaching interventions.

With the use of either conventional means or using imaging techniques like fMRI and EEG as well as eye-trackers and biometrics approaches researchers have published some results in the field of computer programming and especially in the areas of software development, code understanding, identification of novice programmers' needs, cognitive load and debugging.

As described in [31] “subjective judgments in software engineering tasks are of critical importance but can be difficult to study with conventional means”. Researchers argue that imaging techniques can contribute to linking cerebral activity to the cognitive and physical activity of the participants.

Functional magnetic resonance imaging was recently used by researchers to measure program comprehension [32]. Researchers observed a sample of 17 participants, and they identified five areas of the brain that appear to be related to understanding the code of a program. In particular, they identified areas that are related to attention, work memory and language processing. According to the researchers, the results of their study show that fMRI can be used for research in the field of computer programming in conjunction with participants' brain activity in order to improve the training of future programming developers.

The exploration of the neural representations that appeared in the attempt of code comprehension in relation with prose review was the subject of a group of researchers who, using fMRI, studied 29 participants [31]. According to the results of their research, it appears that the neural representations that appeared during code comprehension are different from the neural representations that appear in the study of prose texts. Researchers also argued that “task distinctions are modulated by expertise, such that greater skill predicts a less differentiated neural representation indicating that more skilled participants treat code and prose more similarly at a neural activation level” [31].

In another recent study, the EEG was used to measure programmer expertise. According to the results of their study, it appears that the electrical activity recorded through the EEG can indicate the previous experience of the participants in correspondence with their self-reported experience levels [33]. Researchers linked the cognitive load with the electrical activity of the brain and concluded that it is possible to quantify the performance in computer programming based on the expertise and cognitive requirements of the activities. EEG was also used by other researchers to assess developers' productivity in real time [34].

Heart rate variability was used as a biometric measure by some researchers in a study. The researchers study a team of programmers in order to identify their potential concern when they asked: a) to study a piece of code, b) to check if the code has any mistake, c) to replace a piece of code that is already at the repository with the modified code that they studied in step (a) and (b) [35]. According to the results of their study, biometric measures can contribute in order to predict in a percentage more than 26%, the concerns that some developers can have about the quality and the replacement of a piece of code, which goes beyond classifiers who work with traditional approaches. In a similar direction, researchers used Near Infrared Spectroscopy to measure developers' cerebral blood flow while working on code comprehension tasks with two difficulty levels [36]. Moreover, another researcher investigated the potential of electromyography to measure subvocal utterances and found that this might be used to determine programming task difficulty [37].

Finally, eye-trackers have been used in various studies about computer programming. These studies have concentrated on the type of programming (visual or textual programming), gender [24], the difficulty of developers with specific pieces of code [38] and the understanding of Unified Modeling Language (UML) class diagrams [39]. Research results show that the type of programming and the gender of the programmer influence both the quality and the efficiency of developers.

The studies presented indicate that the use of imaging techniques like EEG and fMRI and biomarkers measures can contribute to research in the field of computer programming. In formal or informal education, these techniques can provide data that can enhance developers' learning and their development. The data can contribute to the understanding of the learners' brain activity and to the modeling of the learning process in order to develop targeted and differentiated curricula in programming.

## 4. Research

Eight (8) computer science students, novice programmers, who were in the first semester of their studies, participated in a field study in order to explore potential differences in their brain activity during programming with a visual programming language versus a textual programming language.

Students were asked to develop two programs (P1 and P2) in two different programming languages (four tasks in total). The order of the programs (P1 and P2) was predetermined, but there was a difference in the order of the programming languages that the students used to develop these programs. As a result, some students develop the program P1 first with a visual programming language and then with a textual programming language and some others develop the P1 program first with a textual programming language and then with a visual programming language with blocks. In order to derive all possible combinations, the same approach was followed for the P2 program (Table 1). This differentiation was chosen for two reasons: (a) in order to be able to compare students' brain activity as they were working on the same program with a visual programming language with blocks compared to a textual programming language; (b) to assess whether students' brain activity is influenced by the order of the languages in which they work.

The EEG imaging method was used to record the brain signal and measure brain activity using a 10/20 system of the standard position of scalp electrodes for a standard EEG record. The BIOPAC data acquisition unit, MP150 and AcqKnowledge 4.3 Software are used for data acquisition, analysis, storage, and retrieval. The EEG electrodes were placed in the C4-P4 scalp position. Silver chloride electrodes were applied following the 10/20 system.

**Table 1.** Tasks order and gender of students.

Student	Gender	Program 1A	Program 1B	Program 2A	Program 2B
S1	M	Scratch	Python	Python	Scratch
S2	M	Scratch	Python	Python	Scratch
S3	F	Python	Scratch	Python	Scratch
S4	M	Python	Scratch	Python	Scratch
S5	F	Python	Scratch	Scratch	Python
S6	F	Python	Scratch	Scratch	Python
S7	M	Scratch	Python	Scratch	Python
S8	M	Scratch	Python	Scratch	Python

The EEG mainly detects the signal of the task performed by the specific brain region where the electrodes are placed in different positions on the scalp [40]. The signal characteristics vary from one state to another. Five major brain waves can be distinguished by their frequency ranges, namely delta (δ) 0.5–4 Hz, theta (θ) 4–8 Hz, alpha (α) 8–13 Hz, beta (β) 13–30 Hz and gamma (γ) 30–128 Hz. The AcqKnowledge software record EEG data, filter the data into the specific bandwidths for Alpha, Theta, Beta and Delta, and display the results both on-line and off-line. On-line calculation channels allow filtering data using FIR and filters to provide optimal signals for analysis and create custom EEG montages. The raw EEG channel is filtered with the use of the FIR with Band Pass option. The EEG is recorded at 1000 samples/sec with a resolution of 12 bits/sample. Then the data is digitally filtered using 1–50 Hz band pass filter.

The data was sampled at 1000 Hz and band-pass filtered FIR with a Hamming window (0.5–30 Hz). The wave was denoised with a hard threshold method fixed in 0.185391. The typical settings were used for the high-pass filter and a low-pass filter: 0.5 Hz and 35 Hz, respectively. The high-pass filter typically filters out the slow artifact, such as electro-galvanic signals and movement artifact, whereas the low-pass filter filters out high-frequency artifacts, such as electromyographic signals.

**Figure 1.**
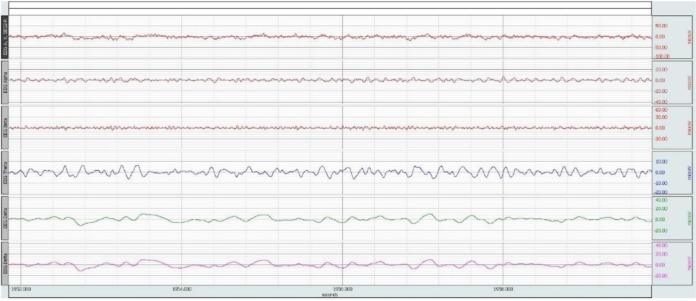
EEG RAW data.

**Figure 2.**
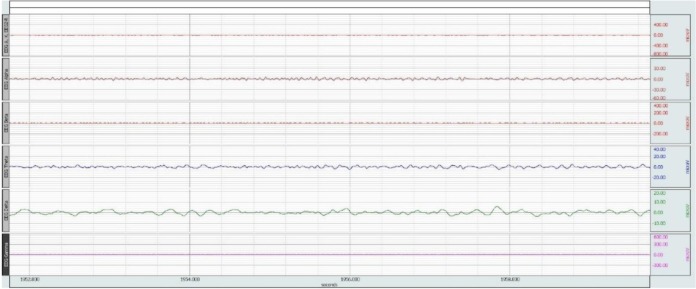
Filtered EEG signal.

For analysis of EEG signal, band pass filter is used, which passes only the data in the specified range and attenuates the rest. The raw data are filtered between ranges 0.5 Hz to 30 Hz. A sample of data before and after the filtering is shown in Figures 1 and 2. The x-axis represents time (second) and the y-axis represents amplitude (microV).

The data were analyzed using the SPSS 17.0 statistical package. Among various statistical measures maximum and minimum value, mean and standard deviation are chosen in order to analyze different mental states of participants while dealing with the programming tasks. In this way, the students' overall brain activity and delta, theta, alpha, beta and gamma brain waves were recorded and analyzed. In particular, this study explores whether for the particular students:

(a) Their brain activity is affected by the range of programming languages they will use to develop a program, i.e., whether their brain activity is influenced by whether they will develop the program first with a visual programming language or first with a textual programming language or vice versa,

(b) the type of programming language (visual or textual) that will be used to develop a program affects their brain activity and

(c) their brain activity is affected as time passes through the development of the two programs (four tasks).

## 5. Research methodology

The research was carried out in the academic year 2017–2018 at the university laboratories and in a properly designed space. A total of eight volunteers computer science students were recruited, three of whom were women. The participants were first-year students, attended the first semester of study and had spent approximately two months since their admission to the university's department. In order to participate in the study, the following criteria were set: a) not to have prior knowledge or experience with the Python and Scratch programming languages, b) to attend two introductory courses in Python and Scratch programming languages (3 hours each), and c) to be healthy adults.

After an individual interview with each student in order to gather some basic demographics data and to investigate the reasons for his/her participation in the study, each student signed the consent form for participation in the research. Subsequently, all students attended the two introductory seminars in Python and Scratch programming languages. The seminars took place on two consecutive days and the facilitator was a researcher from the laboratories.

On the third day, students participated in the experiment. The participants were asked to be psychologically and mentally calm, to be rested and not to drink alcohol before taking this test. Each participant took the test individually in a room with few outside distractions. Initially, volunteers were asked to have a seat in a comfortable position. They were asked if they were feeling comfortable in order to provide any changes to ensure that they were relaxed. Thereafter, they were asked to develop two programs in both the Python and Scratch languages. For the study, the order of languages in which students were asked to develop the programs was differentiated (see Table 1).

Based on the structure shown in Table 1, some students started the first program with Scratch and then they developed the same program with Python, while some other students did the reverse. The same happened with the second program as well as between the two programs. In this way all the possible combinations were created.

At the end of the process, a second round of individual interviews and two focus groups followed in order for the students to express their opinions concerning their preferences with regard to the programming languages they used, the development of programs and the issue of recording their brain activity.

The programs that participants were invited to develop are presented in Table 2.

In this paper we study the overall brain activity of students during their work in each of the four tasks they worked with.

**Table 2.** Programs and tasks.

1A/1B	2A/2B
Develop a program in [A: Python/B: Scratch] that will detect and display the largest among the one hundred numbers that are input through the keyboard. You may assume that all the inputs will be distinct.	Develop a program in [A: Python/B: Scratch] that will: a) detect the largest among the one hundred numbers that are input through the keyboard. b) compute how many times that number occurs in the input c) displays that number and the number of its occurrences.

## 6. Research findings

Students were asked to develop two programs in Python and Scratch languages. They were also given the time to develop all four tasks correctly. For this reason, there are variations in the total time they worked for the tasks.

The data gathered by each student was analyzed with the SPSS 17.0 statistical package. The descriptive analysis of data includes: (a) the student's working time per program (measured in minutes) and (b) the means of EEG and standard deviation of the student's brain activity per program. In the first column there is the student's serial number (S1 to S8), in the second column the language in which he/she worked (Scratch or Python), the task he/she developed in this language (Scratch1 or Scratch2 or Python1 or Python2) and the order of the task (1st, 2nd, 3rd or 4th) (Table 3). The negative value of the means of EEG may reflect a larger cognitive response [41].

**Table 3.** Descriptive analysis.

Student	Program/Task	N	Mean	S.D.
S1	Scratch1_1	10.10	−1.125	306.078
S1	Python1_2	12.66	−1.701	264.993
S1	Python2_3	10.69	−1.129	180.938
S1	Scratch2_4	6.36	−1.045	109.120
S2	Scratch1_1	5.54	−1.110	107.631
S2	Python1_2	1.73	−1.112	40.659
S2	Python2_3	6.55	−1.082	25.615
S2	Scratch2_4	3.15	−1.059	30.022
S3	Python1_1	16.29	−1.308	208.768
S3	Scratch1_2	14.75	−1.219	106.996
S3	Python2_3	11.83	−0.923	187.750
S3	Scratch2_4	11.83	−0.922	187.746
S4	Python1_1	25.05	−1.013	49.171
S4	Scratch1_2	14.67	−1.014	29.407
S4	Scratch2_3	12.58	−1.052	36.252
S4	Python2_4	7.18	−1.047	66.336
S5	Python1_1	10.98	−0.435	277.273
S5	Scratch1_2	3.15	−0.499	183.940
S5	Scratch2_3	9.23	−1.069	184.842
S5	Python2_4	3.31	−0.767	240.303
S6	Scratch1_1	4.47	−1.139	37.423
S6	Python1_2	4.17	−1.096	39.489
S6	Scratch2_3	3.40	−1.056	16.727
S6	Python2_4	2.64	−1.054	32.421
S7	Scratch1_1	19.93	−1.107	61.950
S7	Python1_2	16.45	−1.088	27.634
S7	Scratch2_3	15.86	−1.190	73.911
S7	Python2_4	6.03	−1.091	63.042
S8	Python1_1	10.37	−1.048	96.540
S8	Scratch1_2	8.08	−1.111	49.563
S8	Python2_3	3.83	−1.101	64.392
S8	Scratch2_4	4.14	−1.065	44.523

A first finding is related to the total time (measured in minutes) that the students needed to successfully develop their programs. It seems that six students devoted more time to successfully develop the programs with the first programming language than with the second programming language, regardless of the type of programming language with which they developed the programs (visual or textual). However, there are two students (S1 and S8) where the first one took more time in the first program with the second programming language and the second needed more time in the second program with the second programming language (Table 4).

**Table 4.** Working time.

Student	P1A		P1B		P2A		P2B	
S1	Scratch	10.10	Python	**12.66**	Python	**10.69**	Scratch	6.36
S2	Scratch	**5.54**	Python	1.73	Python	**6.55**	Scratch	3.15
S3	Python	**16.29**	Scratch	14.75	Python	**11.83**	Scratch	11.83
S4	Python	**25.05**	Scratch	14.67	Python	**12.58**	Scratch	7.18
S5	Python	**10.98**	Scratch	3.15	Scratch	**9.23**	Python	3.31
S6	Python	**4.47**	Scratch	4.17	Scratch	**3.40**	Python	2.64
S7	Scratch	**19.93**	Python	16.45	Scratch	**15.86**	Python	6.03
S8	Scratch	**10.37**	Python	8.08	Scratch	3.83	Python	**4.14**

From the above, it appears that the type of programming language does not seem to affect the time that the student will need to develop the required program (provided they know the two programming languages to the same degree). However, it seems that since a student has developed a program with a programming language, writing the same program with another programming language even of a different type requires less time.

Α second finding was related to the brain activity of the students. The analysis shows that two students (S3 and S6) had a constantly increasing brain activity as their time spent engaging in the development of the two programs (Table 5). At the same time, these students showed less time to engage with each task, which means they continuously reduced the time they needed to successfully develop the programs (Table 4). However, the six students did not show specific brain activity as their time spent and they worked with the tasks. At the same time did not show any specific reduction or increase in the time they needed to develop the programs.

**Table 5.** Mean of the brain activity per task.

	P1A		P1B		P2A		P2B	
S1	Scratch	−1.125	Python	−1.701	Python	−1.129	Scratch	−1.045
S2	Scratch	−1.110	Python	−1.112	Python	−1.082	Scratch	−1.059
S3	Python	−1.308	Scratch	−1.219	Python	−0.923	Scratch	−0.922
S4	Python	−1.013	Scratch	−1.014	Python	−1.052	Scratch	−1.047
S5	Python	−0.435	Scratch	−0.499	Scratch	−1.069	Python	−0.767
S6	Python	−1.139	Scratch	−1.096	Scratch	−1.056	Python	−1.054
S7	Scratch	−1.107	Python	−1.088	Scratch	−1.190	Python	−1.091
S8	Scratch	−1.048	Python	−1.111	Scratch	−1.101	Python	−1.065

According to the above finding it seems that students as they develop computer programs do not show a decrease or increase in their brain activity. Also, given that the programming difficulty has increased in the second program, it is interesting that there are variations in brain activity between students. This finding can contribute to highlighting the need for a differentiated learning approach among students while they learn to program.

A third finding is that the student's brain activity was higher when the students were working on the first task than the second one and respectively when they worked on the fourth task compared to the third one. More specifically, the eight students had a higher mean of the brain activity during the development of the last (fourth) task compared to the third one and six of them had a higher mean of the brain activity when working with the first task versus the second one, independently of the programming language they use (Table 6).

**Table 6.** Mean of the brain activity related to the first and last task.

	P1A	P1B	P2A	P2B
S1	**−1.125**	−1.701	−1.129	**−1.045**
S2	**−1.110**	−1.112	−1.082	**−1.059**
S3	**−1.210**	−1.219	−0.923	**−0.923**
S4	**−1.013**	−1.014	−1.052	**−1.052**
S5	**−0.435**	−0.499	−1.069	**−0.767**
S6	−1.139	**−1.096**	−1.056	**−1.054**
S7	−1.107	**−1.088**	−1.190	**−1.091**
S8	**−1.048**	−1.111	−1.101	**−1.065**

This finding links the students' brain activity with the programs they had to develop and, in particular, with the sequence of tasks they had to accomplish. The order of work of each student was determined in advance. They were asked to develop two programs (four tasks) with a different order between the first and the second task and between the third and the fourth task. From the analysis of the data, it appears that the mean of the brain activity of all eight students was higher in the last (4th) task than the third one, which may be related to the educational process itself. One possible explanation is that since it was the last task they had to work with, the mean of their brain activity was higher, because this was the last task remaining in order to complete all the tasks assigned to them. Similarly, six of them showed a higher mean of the brain activity when they worked with the first task versus the second one, regardless of the programming language. It seems that because with the first task starts simultaneously the EEG recording, the students had a more intense brain activity when they worked with it.

## 7. Discussion

The results of this study attempt to contribute to the discussion on the importance of recording brain activity in order to strengthen teaching interventions and develop appropriate learning pathways. The results show that there were differences in participants' brain activity when developing computer programs. This element leads to the finding that, like the learning of a second foreign language [42], learning programming is a complex, dynamic, open, self-organized and adaptable subject and therefore requires a differentiated approach depending on the learning skills and different cognitive abilities of individuals. The need for a differentiated learning approach to enhance different brain activities during programming leads to the development of appropriate learning paths to differentiate learning processes and learning contents [43]. The above are also related to the results of [44]. The researchers, using EEG, demonstrated that the differential learning approach stimulates the somatosensory and motor system and involves more areas of the cortex in relation to recurrent learning.

Of particular interest is the importance of engaging individuals with textual and visual programming languages. From this research it appears that program development is not affected by the type of programming language that the programmer will use. In this way, the optimization of the programming language learning process can be enhanced by following a nonlinear learning pathway, which can lead to better results in lessening students' aversion from deepening into programming.

Another interesting feature is the issue of cognitive enhancement of students, which can be take place in the cognitive processes that are performed during computer programming and the modification of the hierarchy of the tasks in order to make them more appropriate.

## 8. Limitations of the study

The study involves statistical measurements and people, and it is therefore necessary to recognize that there are some limitations. This research involved a small number of participants and a fixed number of tasks. This model may be restrictive and for this reason it is important to repeat this study as an independent research to confirm the results. Participants are volunteers and elected after an open call. In addition, another limitation of this research is the selected tasks. Although an attempt was made in order to select tasks that may be considered representative of student level and level of difficulty, it remains possible that different tasks may result in different results.

In addition, security issues and ethical issues have as a result (as in all EEG data) some constraints, since research has been conducted in an office/lab environment instead of a site where developers could work independently. An inexpensive device (a low-cost device) was used for the survey. This option, although restrictive, may favor expanding research with other devices that have more capabilities.

## 9. Future research

In this study we analyzed the mean of the brain activity of the participants as they developed programs in two programming languages belonging to two different types of programming (textual and visual programming).

Research data includes measurements for alpha, beta, gamma, delta and theta activity which will be further studied. In addition, it is interesting to study brain activity at the time of program execution, where a first check take place in order to execute the program. Currently, it is interesting to study the participants' brain activity if their programs have syntax errors or if their programs are executed and have logic errors.

The repetition of this research by the simultaneous study of a) neurobiological mechanisms that provide evidence for student anxiety and b) specific biomarkers [45] can contribute to the development of a richer image of learning and programming training.

## 10. Conclusion

This study observed eight first-year students, novice programmers; the students' brain activity was recorded as they developed programs in two programming languages that belong to two different types of programming (textual and visual). Their brain activity was recorded using the EEG imaging method. The analysis has shown that programming is a non-linear and simultaneously dynamic process characterized by complexity and requires a differentiated approach based on learning skills, cognitive abilities and biometric study of individuals.

The primary goal of neuroscience research is to carry out applied research that will generate data that will contribute to the field of education. The results of this research can then contribute to the debate on improving and enhancing educational processes. Predicting human behavior is an interesting challenge. According to recent US research initiatives “An overarching goal...is to expand our understanding of the impact of an individual's current state on future behavior and to increase predictability of the individual or team by (1) gaining access to covert mental activity inaccessible through common means, or (2) biasing the individual toward more reliable behavior. This mental activity includes but is not limited to perception, attention, decision-making, and communication.” [46].

The objective of this study was to contribute to the open research debate on increasing the predictability of individual or group behavior and to contribute to the programming education and the recognition of appropriate learning pathways through the study of the observed brain activity and according to the trainees' profile and educational framework.
